# Striking the Balance: GLP-1/Glucagon Co-Agonism as a Treatment Strategy for Obesity

**DOI:** 10.3389/fendo.2021.735019

**Published:** 2021-09-08

**Authors:** David C. D. Hope, Matthew L. Vincent, Tricia M. M. Tan

**Affiliations:** Division of Diabetes, Endocrinology and Metabolism, Department of Metabolism, Digestion and Reproduction, Imperial College London, London, United Kingdom

**Keywords:** glucagon, GLP-1, obesity, co-agonist, weight loss

## Abstract

Obesity and Type 2 diabetes represent global health challenges, and there is an unmet need for long-lasting and effective pharmacotherapies. Although long-acting glucagon-like peptide-1 (GLP-1) analogues are now in routine use for diabetes and are now being utilised for obesity *per se*, the need for ever better treatments has driven the development of co-agonists, with the theoretical advantages of improved efficacy by targeting multiple pathways and reduced adverse effects. In this review, we highlight the past and present progress in our understanding and development of treatments based on GLP-1/glucagon co-agonism. We also reflect on the divergent effects of varying the GLP-1:glucagon activity and ratio in the context of pre-clinical and human clinical trial findings. In particular, the multiple metabolic actions of glucagon highlight the importance of understanding the contributions of individual hormone action to inform the safe, effective and tailored use of GLP-1/glucagon co-agonists to target weight loss and metabolic disease in the future.

## Introduction – Glucagon as an Anti-Obesity Agent

Obesity is a leading cause of global morbidity and death. It is a driver of multiple co-morbidities such as type 2 diabetes (T2D), non-alcoholic fatty liver disease (NAFLD), hypertension, hypercholesterolaemia, cardiovascular disease and cancer (1). In 2016, 1.9 billion adults were classified as overweight and 650 million as obese according to the World Health Organisation in 2016. The current COVID-19 pandemic has also highlighted the strong link between obesity and poorer outcomes (2, 3). There is a growing health and socioeconomic burden of obesity, with an increasing demand for effective anti-obesity drugs ideally comparable to bariatric surgery, the current gold-standard for obesity treatment which offers highly effective, long-lasting and life-extending results (4). The gut hormone glucagon-like peptide-1 (GLP-1) and its analogues, which have been in clinical use for diabetes for over a decade, have useful appetite-suppressive effects and are now licensed for obesity. Despite the undeniable success of the GLP-1 analogues, there remains a ‘gap’ between the efficacy of GLP-1 analogues and that of bariatric surgery. To plug this gap, researchers have pursued the ‘co-agonist’ strategy by combining GLP-1 with related hormones from the proglucagon family and related peptides, including GIP and glucagon itself (5). By combining hormones in this way, the dose of individual hormones can be reduced, widening the therapeutic window and avoiding toxicity. In this mini-review, we highlight past and present progress in the translational research of GLP-1 and glucagon co-agonism. We also highlight the importance of striking a balance between GLP-1 and glucagon agonism, to allow for maximal drug efficacy whilst minimising potential risks.

## Oxyntomodulin, a Natural GLP-1 and Glucagon Co-Agonist

**Figure 1:**
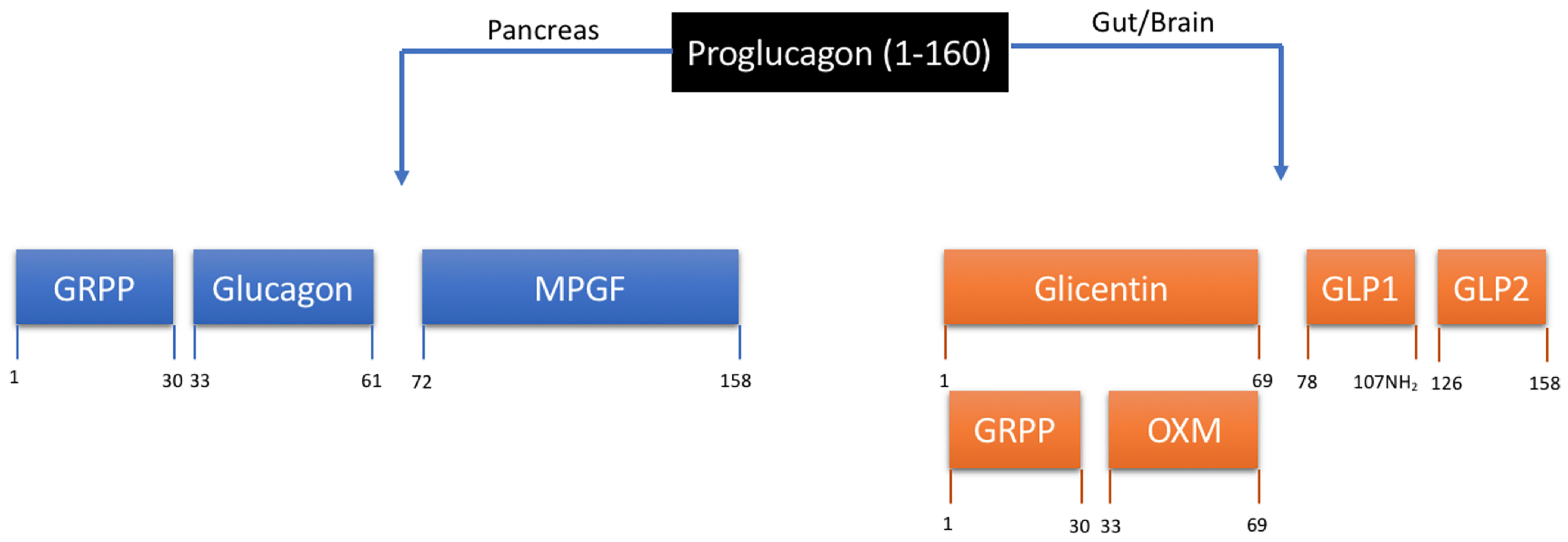
Tissue-specific processing of proglucagon. Proteolytic cleavage is tissue specific and regulated by prohormone convertases (PC) 1 and 2. In the pancreas, PC2 results in the formation of glucagon, glicentin-related pancreatic peptide (GRPP) and the inactive fusion protein major proglucagon fragment (MPGF). In the gut and brain, PC1/3 results in the formation of glicentin, GLP-1 and GLP-2. Glicentin is further processed to form GRPP and oxyntomodulin (OXM). Numerical annotations represent amino acid positions within the 160 amino acid proglucagon peptide.

The journey to discovery of the glucagon family of peptides and an endogenous GLP-1/glucagon co-agonist, oxyntomodulin (OXM) is an example of a concerted effort from many dedicated research groups. A pivotal point early on in this research was the use of the known peptide sequence of glucagon to facilitate the discovery of other ‘glucagon-like peptides’ in the gastrointestinal tract with the help of the radioimmunoassay method (6, 7). The search for ‘glucagon-like reactivity (GLI)’ in the gut revealed a partial peptide sequence for a peptide named ‘Glicentin’, later to be fully characterised as a 69-amino acid peptide containing a 30-amino acid ‘Glicentin-related pancreatic polypeptide’ (GRPP), the full sequence of glucagon, and an 8-amino acid c-terminal extension (8–10). The 8-amino acid extended glucagon fragment, ‘Glucagon-37’ was isolated from porcine jejuno-ileum, characterised and shown to be the bioactive ‘enteroglucagon’ due to its ability to bind to and stimulate glucagon receptors in liver membrane extracts (11–13). Due to the potent effect on oxyntic cell signalling, bioactive enteroglucagon/Glucagon-37 was named oxyntomodulin (14). As the primary structure of glicentin was discovered, evidence also emerged for post-translational processing of proglucagon to form glucagon and glicentin related pancreatic peptide, secreted ‘synchronously’ from the pancreatic alpha cell (10). Beyond the protein-based methodologies used to characterise glicentin, GRPP and oxyntomodulin, the increased capability to sequence genes at the time led to the first sequence of mammalian preproglucagon (15). This revealed two further glucagon-like polypeptides, now known as GLP-1 and GLP-2, and confirmed earlier reports suggesting that the MW of proglucagon is much larger than that of glicentin alone (16). The post-translational processing of pro-glucagon is now known to be differentially regulated in pancreas and gut (17, 18). Alternative processing of proglucagon leads to the formation of glucagon, GRPP and major pro-glucagon fragment (MPGF) in the pancreas, whereas in the gut and brain glicentin, GRPP, oxyntomodulin, GLP-1 and GLP-2 are formed (**Figure 1**). The endogenous gut hormone oxyntomodulin is therefore formed through specific splicing of the proglucagon gene and includes the full sequence of glucagon along with the 8-amino acid sequence named IP-1. This process occurs in the intestinal L cells of the gastrointestinal tract leading to the co-secretion of GLP-1 and oxyntomodulin in response to nutrient sensing (19, 20).

Whilst much attention was focused on GLP-1 in the 1980s and 90s, following the discovery of its incretin effect in humans (21), little was known about the physiological role of oxyntomodulin in humans at the time (21, 22). Infusion studies in humans demonstrated a potent effect of high levels of OXM infusion on gastric emptying (23). Furthermore, changes in OXM were observed following intestinal bypass surgery suggesting anatomical changes influenced intestinal secretion of the peptide (24, 25). In the early 2000s, its potential role in weight loss was investigated following research showing that GLP-1 and glucagon inhibit food intake when administered intracerebroventricularly (ICV) in rodents (26, 27). ICV injection of OXM led to a significant food intake reduction up to 4 hours after injection, comparable to GLP-1 infusion (28). This effect was inhibited by the GLP-1 antagonist exendin (9–39), suggesting that OXM may act through the GLP-1 receptor (GLP-1R) to regulate food intake. Daily ICV injections of OXM over 7 days led to food intake reduction and increased weight loss compared with saline treated controls (29). Importantly, OXM-treated rats had increased weight loss compared to pair-fed controls and this was associated with a 0.5°C increase in core body temperature during the seven-day treatment period, suggesting for the first time an energy expenditure effect of OXM that is independent of its anorectic effects. Peripherally administered OXM led to a dose-dependent food intake reduction and increased weight loss in rats, and these animals lost significantly more body weight and white adipose tissue than pair-fed controls. ICV injection of exendin-9-39 attenuated the anorectic effect of peripheral OXM, further suggesting that OXM-mediated anorexia is produced through a central GLP-1R-dependent mechanism (30).

Concurrent to the pre-clinical studies, the first human study of oxyntomodulin was carried out in a small double-blind placebo-controlled crossover study (31). Thirteen healthy participants were given intravenous infusions of OXM at a dose of 3 pmol/kg/min for 90 minutes, with matched controls being given saline. During this infusion period, the *ad-libitum* food intake was significantly reduced in participants receiving OXM compared to saline control and a cumulative caloric intake reduction was observed up to 12 hours later. Later, a 28-day randomized controlled trial examined the effect of prolonged OXM injections on body weight loss and energy intake. Healthy overweight volunteers were randomised to receive pre-prandial subcutaneous injections with either saline or 400 nmol OXM three times daily, coincident with meals, for 4 weeks. OXM-treated subjects were found to have decreased energy intake on Days 2 (660 *vs* 508 kJ) and 29 (711 *vs* 428 kJ). At the end of the 28-day period, study subjects experienced significant weight loss (2.3 kg *vs* 0.5 kg) compared to saline treated controls in addition to concurrent changes in leptin and adiponectin suggesting reduction in adiposity (32).

## Oxyntomodulin Activates Both GLP-1 and Glucagon Receptors Controlling Energy Balance and Glycaemia

Given OXM contains the entire glucagon sequence, it is no surprise that this peptide activates the glucagon receptor (13, 33). However, the potent effect on reducing weight gain in pre-clinical studies led to research efforts to determine the CNS binding site of OXM. OXM was shown to increase cAMP production in Baby Hamster Kidney (BHK) cells transfected with rodent GLP-1R and GCGR, suggesting meaningful signalling through both receptors but was less potent than Exendin-4 at the GLP-1R (34). The anorectic effects of centrally administered OXM were also abolished in *Glp-1r^-/-^* knock-out mice and not in *Gcgr^-/-^* mice, suggesting that the anorectic effects of OXM are mediated through GLP-1R. Consistent with these findings, previous *in vitro* studies showed that OXM is a full agonist at the GCGR and GLP-1R but is 3-fold less potent at the GCGR than native glucagon and 100-fold less potent at the GLP-1R than native GLP-1, in terms of activating cAMP accumulation (13).

Despite convincing data suggesting the central role of GLP-1R signalling in the food intake reduction effects of OXM, there were still some unexplained effects of the gut hormone including increased energy expenditure, suggesting a GLP-1R independent mechanism of action (35). The stimulatory effect of glucagon on thermogenesis in brown adipose tissue *in vitro* in addition to enhanced metabolic rate in humans had been previously shown (36, 37). Kosinski and colleagues constructed a variant of OXM, OXMQ3E, that was unable to activate the GCGR by changing the third amino acid residue from neutral glutamine to acidic glutamate (38). OXMQ3E produced less weight loss compared with OXM in diet-induced obese (DIO) mice despite similar food intake reduction. GCGR antagonism also reduced the weight loss effect of OXM, suggesting a role for GCGR mediated energy expenditure (38). Moreover, the OXMQ3E peptide was unable to produce any weight loss in *Glp1r*^-/-^ mice, suggesting that both GLP-1R and GCGR are required for weight loss to occur. Importantly, OXM treatment led to several beneficial metabolic effects not seen in OXMQ3E treated mice including a decrease in plasma triglycerides and plasma cholesterol in addition to an increase in adiponectin. More recently, metabolic cage studies have confirmed that the increased oxygen consumption and energy expenditure effect of OXM is mediated through glucagon receptor signalling (29, 39, 40).

To characterise the effects of OXM on glucose metabolism, Du and colleagues performed hyperglycaemic clamp studies in mice treated with OXM and OXMQ3E (41). Both OXM and OXMQ3E improved glucose tolerance when given to DIO mice. The authors found the glucose infusion rate (GIR) during the clamp study decreased with OXM compared with OXMQ3E in wild-type mice, likely due to increased hepatic glucose output from GCGR agonism with OXM. Therefore, concurrent agonism at the GLP-1R and GCGR is important for the glucose-lowering effect of OXM: GLP-1R activation offsets the hyperglycaemia associated with GCGR activation. As we will discuss later, this further justified the development of synthetic GLP-1/glucagon co-agonists as promising weight loss therapeutics, particularly in the context of avoiding unwanted hyperglycaemia in obesity-associated type 2 diabetes.

## Rationale for GLP-1/Glucagon Combination Therapy for Weight Loss

Although significant progress was made in understanding the potential use of the endogenous GLP-1/glucagon co-agonist OXM as a weight loss therapeutic in the early 2000s, evidence from various pre-clinical and human studies prior to this demonstrated the distinct mechanisms of weight loss afforded by these hormones. GLP-1, secreted from the intestinal L cells postprandially, acts both centrally and peripherally to exert its anorectic and metabolic effects. ICV GLP-1 reduces food intake in rodents, and this effect is blocked with Exendin (9–39). It is now known that GLP-1 receptors are widely expressed in the hypothalamus, hindbrain and amygdala with a neuronal link between the periphery and CNS to regulate GLP-1 action (42). In addition to the effect on food intake reduction and glucose-stimulated insulin secretion, GLP-1 also acts to delay gastric emptying therefore aiding satiety and glycaemic control (42). Given at higher doses than in diabetes treatment, GLP-1 analogues are now routinely licensed for the treatment of obesity, for example high-dose Liraglutide 3 mg daily (43) and Semaglutide 2.4 mg weekly (44). However, despite the progress in the development of GLP-1 analogues, the overall efficacy of GLP-1 analogues for weight loss is still limited by gastrointestinal side effects, in particular nausea at higher doses (45).

Glucagon is typically stimulated by the fall in insulin during fasting in concert with low glucose levels, and responds to hypoglycaemia by mobilising hepatic glucose through the stimulation of glycogenolysis and gluconeogenesis (46). In T2D, loss of the suppressive effects of glucagon and insulin is associated with hyperglucagonaemia and this is thought to contribute to hyperglycaemia (47–50). Based on evidence for the contribution of hyperglucagonemia to hyperglycaemia in T2D, research demonstrated the use of glucagon antagonism to improve glycaemic control. In their seminal study, Peterson and Sullivan utilised a novel non-peptide glucagon receptor antagonist (Bay 27-9955) in healthy, lean males to investigate effects on glycaemia (51). Following oral administration of Bay 27-9955 at two doses, a significant blunting of acute hyperglycaemia was observed following a glucagon infusion, in the absence of clinical side effects. Following the promising results of Bay 27-9955, several glucagon receptor antagonists have been developed however when administered over a longer period, have been met with several adverse effects including increased plasma alanine aminotransferase (ALT), LDL-cholesterol in addition to increased blood pressure and body weight (52). Furthermore, treatment with the glucagon antagonist, LY2409021 over 6 months led to a significant increase in both ALT and hepatic fat fraction (HFF) compared to sitagliptin and placebo groups (53). The development of hepatic steatosis is likely due to blockade of glucagon’s lipolytic properties in the liver, therefore making GCGR antagonism an untenable strategy. Despite its glucose mobilising effect, the broad catabolic and thermogenic nature of glucagon receptor agonism adds to its attractive portfolio as a weight loss therapeutic. The enhanced metabolic rate observed with glucagon administration was shown early on in rodents and humans (36, 40). Glucagon-induced brown adipose tissue (BAT) thermogenesis has been demonstrated in rodents (37, 54). However this seems to be species specific: BAT activation is not observed in humans with a glucagon infusion (55). Futile cycling of glucose has also been suggested to confer glucagon’s energy expenditure effects, whereby glucagon stimulates opposing pathways of hepatic glucose production and consumption (56). Circulating FGF-21 has also been implicated in energy expenditure effects with chronic glucagon agonism, as *Fgf21*^-/-^ knock-out mice are protected from these effects (57). Despite several postulated theories of glucagon induced energy expenditure, the mechanism is likely multi-faceted and the precise contribution to these facets remains uncertain (58). Glucagon is also known to act peripherally to enhance lipolysis in white adipose tissue and improve whole body lipid metabolism (59, 60). In particular, glucagon has firmly been demonstrated to enhance hepatic lipid metabolism, Hepatic GCGR agonism leads to upregulation of lipid catabolism pathways in the hepatocyte where a number of key regulatory transporters and enzymes facilitate beta oxidation of fatty acids (61). Furthermore, exogenous administration of glucagon was shown early on to inhibit food intake in humans and rodents (62, 63). Interestingly, this effect seems to be mediated through hepatic glucagon signalling, as infusion into the portal vein induced a satiating response whereas infusion into the inferior vena cava did not (64). The combination of GLP-1 and glucagon administration in rodents has been shown to increase c-Fos expression in appetite regulating centres and polypharmacy with these hormones leads to a synergistic effect on food intake reduction over single hormone administration (65).

Given the pre-existing data on GLP-1 and glucagon, we designed a double-blinded randomised cross-over study, in which we gave volunteers who were overweight short-term intravenous infusions of glucagon (50 ng/kg/min or 14 pmol/kg/min), GLP-1 (0.8 pmol/kg/min), combination of glucagon + GLP-1 at the same doses, or placebo over 45 minutes, and demonstrated that there was a significant increase in resting energy expenditure with glucagon alone which was preserved with the combination of glucagon + GLP-1, whereas GLP-1 did not affect energy expenditure (66). As expected, glucagon infusion caused an increase in glucose which was largely neutralised by co-infusion with GLP-1. In a follow up study with lower, sub-anorectic doses of GLP-1 (0.4 pmol/kg/min), glucagon (2.8 pmol/kg/min), GLP-1 + glucagon combination at the same doses, or placebo for 120 minutes we showed that there was a 13% decrease in food intake after the combination, with the individual infusions having no significant effect (67), further supporting the concept of co-agonism with these hormones. Another study did not show any differences with the hormone combination on glucose and food intake reduction or enhanced energy expenditure but used far lower doses of GLP-1 (1 pmol/kg/min) and glucagon (0.86 pmol/kg/min) (68). Overall, these physiological studies support the notion that GLP-1 and glucagon possess dose-dependent synergism, leading to enhanced suppression of food intake, plus increased resting energy expenditure.

## Pre-Clinical Studies of Synthetic GLP-1/Glucagon Co-Agonists

Concurrent to research investigating combination treatments with individual GLP-1 and glucagon infusions, efforts were also focused on optimising the peptide chemistry of native oxyntomodulin, glucagon or GLP-1 in view of designing receptor potent and long-acting synthetic GLP-1/GCG co-agonists. While the results from studies using native OXM in rodents and humans were promising, the short half-life *in vivo* and the large amount of peptide required to produce an effect made it a poor choice as a treatment in humans. As such, synthetic GLP-1/GCG co-agonists resistant to dipeptidyl peptidase-4 (DPP-4) proteolysis became an attractive target for anti-obesity treatments. Due to the uncertainty regarding the optimum balance of GLP-1R and GCGR agonism in a unimolecular co-agonist, development of novel peptides varied from sequence modification and enhancing stability of native OXM, sequence modification of the glucagon peptide to confer increased GLP-1R potency or modification of GLP-1 peptide to confer increased GCGR potency; the natural advantage of this concept was due to the sequence similarity across the glucagon family of peptides.

By modifying the OXM peptide sequence with an amino acid-peptide substitution at position 2 to prevent DPP-4 action, in addition to a cholesterol-peptide conjugate, Pocai and colleagues were the first group to show that synthetic dual-agonists were an effective treatment strategy in an animal model. They showed that administration of their modified long-acting OXM peptide led to a decrease in food intake and increased weight loss in addition to improved metabolic profile, superior to GLP-1 alone over 14 days in diet-induced obese (DIO) mice (69). Further studies by a variety of groups demonstrated structural modifications of OXM to enhance the longevity of the native peptide through modification with polyethylene glycol (PEGylation), fatty acid conjugation and amino acid substitutions (70–73). By modifying the primary sequence of OXM and linking to the constant region of human IgG4, Jung and colleagues demonstrated their peptide HM12525A had a potent and balanced activity at both GLP-1 and glucagon receptors *in vitro* and led to body weight loss of 30% in DIO mice over 14 days compared to liraglutide treated mice (74). Enhanced energy expenditure was also observed with HM12525A in addition to reduced adiposity and improved liver function in a NASH model in *db/db* mice. HM12525A has been variously re-designated as JNJ-64565111 and now efinopegdutide, and this drug has been taken forward into Phase 2 trials (see below).

Day and colleagues were the first group to use a series of modifications to the C-terminal portion of the glucagon sequence to increase GLP-1R potency, combined with PEGylation to generate two PEGylated peptides with adequate co-agonist properties, one unbalanced towards GLP-1R, and the other a ‘near-balanced’ agonist at both GLP-1R and GCGR. The near-balanced peptide was shown to be ~2-fold less potent at GLP-1R and ~10 fold less potent at GCGR than native ligands in cAMP synthesis (75). When given to DIO mice, both peptides led to significant weight reduction, improved glucose tolerance, increased energy expenditure, and reductions in plasma cholesterol and liver steatosis. These effects were more dramatic with the balanced agonist and interestingly the beneficial metabolic effects occurred with no change in oral nutrient intake in peptide *vs* control groups. This study demonstrated the importance of combined GLP-1R and GCGR signalling, as administration of the balanced co-agonist to *Glp1r*^-/-^ mice led to hyperglycaemia, further demonstrating the necessity of GLP-1R and GCGR co-agonism to minimise this predicted side-effect. The same group further assessed various ratios of GCGR to GLP-1R potency of their peptides, on the extent of weight loss while minimising hyperglycaemia (76). Importantly, the authors demonstrated that the peptides which were most able to produce weight loss without hyperglycaemia demonstrated balanced potency at the GLP-1 and glucagon receptors.

Other groups have also modified the primary sequence of glucagon to confer increased GLP-1R potency. MEDI0832 (now known as cotadutide) is a balanced co-agonist based on the peptide sequence of glucagon, modified at specific amino acid positions in addition to a palmitic fatty acid side chain to prolong activity. The novel peptide is biased towards GLP-1R agonism versus GCGR with a 3-4 fold reduced potency at the GLP-1R compared to native GLP-1 and around an 8-fold reduced potency at the GCGR compared to native glucagon. Chronic daily administration of cotadutide over 27 days in obese mice was associated with food intake reduction, decreased adiposity, improved fasting glucose, and increased energy expenditure. Consistent with previous studies, cotadutide outperformed GLP-1 alone (40 nmol/kg/day liraglutide) in achieving maximal weight loss in obese mice, and this was attributed to the increased energy expenditure (77). Cotadutide has been taken forward in clinical trial development (see below).

An alternative approach in the design of effective synthetic co-agonists has been to modify GLP-1 analogues to confer increased GCG activity. By engineering the C-terminal portion of Exendin-4 to include amino acid sequences from glucagon in addition to a fatty acid side chain, Evers and colleagues developed a potent balanced co-agonist ‘peptide 14’. This was shown to be around 10 times less potent at the human GLP-1R and approximately equivalent potency at GCGR in comparison to the native peptides at eliciting cAMP response *in vitro*. Daily administration of peptide 14 in DIO mice over 32 days led to a 30% body weight loss above that seen for liraglutide alone at 15% body weight loss. In *db/db* mice, peptide 14 prevented a 1.5% increase in HbA1C over 32 days seen in the vehicle control treated group (78). Based on these pre-clinical findings, a lead candidate, SAR425899 with similar receptor potencies was taken forward in Phase 1 and Phase 2 trials (see below).

Novel strategies have also been employed to enhance the pharmacokinetic profile of GLP-1/glucagon analogues in view of prolongation of drug effect. Recently, a surfactant conjugated co-agonist peptide ‘17’ was shown to have a half-life of 52 hours *in vivo* and at a higher dose led to 40% body weight loss in obese rats over 27 days (79). This has subsequently been taken forward into Phase 1 clinical trials as ALT-801 (**Table 1**).

**Table 1:** Current glucagon containing anti-obesity drugs in development.

Drug	Receptor target	Administration	Sequence modified	Receptor potency at human GLP1R and GCGR compared with native hormones. (Based on *in vitro* cAMP EC50 data)		GLP-1/GCGR ratio	Status	Ref
				GLP1R	GCGR			
Cotadutide	GLP-1/glucagon	sc daily	Glucagon	3-4 fold lower	~8 fold lower	5:1	In Phase 2 for kidney disease	NCT04515849 (77)
SAR425899	GLP-1/glucagon	sc daily	GLP-1	~1:1	~13 fold lower	5:1	Discontinued	(80)
MOD-6031	GLP-1/glucagon	sc weekly	OXM	?	?	?	Discontinued	NCT02692781
G3215	GLP-1/glucagon	sc continuous	OXM	~1:1 fold lower	~1:1 fold lower	1:1	Phase 1	NCT02692040
NNC9204-1177	GLP-1/glucagon	sc weekly	?	?	?	?	Discontinued	NCT03308721
Efinopegdutide	GLP-1/glucagon	sc weekly	OXM	~3 fold lower	~3 fold lower	1:1	Phase 2 for NAFLD	NCT03486392 (81)
BI 456906	GLP-1/glucagon	sc weekly	Glucagon	?	?	?	Phase 2	NCT04153929
OPK-88003/TT401	GLP-1/glucagon	sc weekly	OXM	?	?	?	Discontinued	NCT03406377
MK-8521	GLP-1/glucagon	sc daily	?	?	?	?	Discontinued	NCT02492763
LY3305677	GLP-1/glucagon	sc weekly	OXM	?	?	?	Phase 1	NCT03928379
ALT-801	GLP-1/glucagon	sc weekly	GLP-1 and glucagon	?	?	?	Phase 1	NCT04561245 (79)
JNJ-54728518	GLP-1/glucagon	sc daily	OXM	?	?	?	Phase 2	NCT03486392
HM15211	GLP-1/GIP/Glucagon	sc weekly	Glucagon	?	?	?	Phase 2	NCT04505436
NN9204-1706	GLP-1/GIP/Glucagon	sc daily	?	?	?	?	Phase 1	NCT03661879
SAR441255	GLP-1/GIP/Glucagon	sc daily	?	?	?	?	Discontinued	NCT04521738
LY3437943	GLP-1/GIP/Glucagon	sc weekly	?	?	?	?	Phase 1	NCT04143802

?, unknown. NCT numbers from ClinicalTrials.gov.

## Lessons From GLP-1/Glucagon Co-Agonists in Clinical Development

The first GLP-1/glucagon co-agonist to advance to human clinical trials was cotadutide (MEDI0832). In a randomised, placebo-controlled double blinded phase 1 study of ascending single doses in healthy overweight humans, cotadutide was shown to be safe and, in common with the GLP-1 analogues, to be associated with dose-dependent gastrointestinal adverse events especially nausea and vomiting. As an exploratory outcome, single doses of cotadutide led to dose-dependent improvement in glucose excursions post meals within 24 hours and food intake reduction from a single dose of 100 μg. Doses as low as 10 μg had a beneficial effect on post meal glucose excursions and there was no evidence of glucagon-induced hyperglycaemia (82). In a combined multiple ascending dose (MAD) and Phase 2a study in people with type 2 diabetes over 41 days, the safety of cotadutide was confirmed (83). Over 41 days, daily doses of cotadutide of up to 200 μg led to improved glucose AUC_0-4h_ after a mixed meal in comparison to placebo as well as fasting and post prandial glucose levels. Cotadutide also led to 2.1 kg body weight loss relative to placebo. In a separate follow up Phase 2a study over 49 days, the mechanism of improved glycaemia was shown to be a combination of enhanced insulin secretion and delayed gastric emptying (84).

An important exploratory outcome measured in the initial 41-day Phase 2a study was the effect of cotadutide treatment on liver fat reduction as measured by MRI, with a 39.12% reduction was observed in the treatment group compared to 19.51% in placebo group (83). This significant reduction in liver fat content with cotadutide is likely to be due to hepatic glucagon signalling and subsequent upregulation of fatty acid oxidation. In a Phase 2b study in people with type 2 diabetes and obesity over 54 weeks, treatment with cotadutide at doses of 100-300 μg daily improved some non-invasive markers of NAFLD such as transaminase levels, the FIB-4 index, fatty liver disease fibrosis score (NFS) and fatty liver index (FLI) but fatty liver disease was not assessed directly with liver biopsy. This study also included an open-label comparator arm where participants took liraglutide 1.8 mg. Improvements in HbA1c were shown to be similar with reductions of 1.03-1.19% with cotadutide *vs* 1.17% with liraglutide. With respect to body weight, cotadutide at 200 μg led to weight loss of 3.22 kg on average versus 3.33 kg with liraglutide, but the 300 μg dose led to weight loss of 5.02 kg albeit with far higher gastrointestinal adverse event rates than liraglutide (85).

Although cotadutide has progressed well through clinical trials, other GLP-1/glucagon co-agonists have shown mixed results. A recent Phase 2 randomised placebo-controlled trial tested efinopegdutide in people with obesity over a 26-week period, where participants were randomly assigned weekly treatment with either placebo, 5 mg, 7.4 mg, 10 mg efinopegdutide, or 3 mg liraglutide daily. Participants given efinopegdutide showed a dose-dependent increase in body weight loss of 6.7 to 10.0% (placebo subtracted). Participants taking liraglutide achieved a placebo subtracted weight loss of 5.8% in line with clinical experience. Although there was no significant improvement in glycaemia with efinopegdutide, this was explicable given that the participants had normal glycaemia at baseline. However, up to 89% of participants taking efinopegdutide experienced gastrointestinal adverse events (mostly nausea, vomiting and diarrhoea) relative to 28% taking placebo and 60% taking liraglutide (86). This may be because the efinopegdutide arms did not have a dose titration phase unlike the liraglutide arm (86). The original development partner, Janssen Pharmaceuticals, has handed back the license for efinopegdutide to the original developer, Hanmi Pharmaceutical, but the license has been taken up by Merck to be developed as a once weekly treatment for NAFLD.

SAR425899 (Sanofi) is another example of a GLP-1/glucagon co-agonist which has been tested in Phase 1 trials. When tested as a once-daily injection in single and multiple doses varying up to 0.18 mg in healthy normal to overweight volunteers, gastrointestinal adverse events (nausea, diarrhoea, constipation, vomiting) were encountered, but the drug was described as well tolerated. The multiple-dose regimen, given for up to 4 weeks, led to a dose-dependent weight loss between 2.87 and 5.46 kg, compared to 2.37 kg for placebo. In a small group with T2D, SAR425899 improved fasting glucose and glucose tolerance after a mixed meal (80). Results from Phase 2 trials were subsequently halted due to excessive rates of gastrointestinal adverse events leading to participant withdrawals. It is unlikely therefore this drug will proceed further in development.

Several other multi-agonists capable of co-agonism of the GLP-1 and glucagon receptors are currently in development including BI 456906 (Boehringer Ingelheim), LY3305677 (Lilly) LY4347943 (Lilly), JNJ-54729518 (J&J), HM15211 (Hanmi), NNC9204-1706 (Novo), Alt-801 (Altimmune) and G3215 (Imperial College/Zihipp Ltd.) – see **Table 1**. Led by the results of cotadutide, GLP-1/glucagon co-agonists have been shown to have promising weight loss and glycaemic effects in these early phase clinical results however data from later phase clinical trials are expected later this year/early next year. Data from longer-term treatment will therefore be eagerly awaited to determine the extent of body weight loss and metabolic outcomes and whether this is comparable to the latest GLP-1 mono-agonist therapies.

## Striking the Balance of GLP-1 and Glucagon Agonism to Minimise Potential Risks

Based on outcomes from safety and pharmacokinetic studies with GLP-1 analogues, the most common side effect observed with GLP-1 agonism is dose-dependent nausea and vomiting (45). A theoretical advantage of a co-agonist approach is the ability to reduce the dose of GLP-1 whilst also enhancing glucagon’s weight loss effects. As the current development leader, cotadutide has still not been able to escape the spectre of dose-dependent gastrointestinal adverse events (82). Fortunately, a tolerated treatment dose window of 150 μg daily or less of cotadutide was associated with fewer adverse effects and this facilitated its progress to Phase 2 where its marked beneficial metabolic effects were observed.

Even with careful engineering of receptor balance, potency and pharmacokinetics, problems may still crop up, as illustrated by SAR425899’s unexpected development failure due to excessive gastrointestinal adverse events during its Phase 2 trials. A follow up study using radio-ligand PET technology to measure receptor occupancy demonstrated a high degree of GLP-1R occupancy but no detectable GCGR occupancy suggesting that *in vivo* SAR425899 may be acting in effect as a GLP-1 analogue and not a co-agonist (87). The higher frequency of gastrointestinal side effects also observed with efinopegutide in Phase 2 trials may also be explained by a relatively high receptor potency of at GCGR and GLP-1R, within 3-fold of the native ligands (81). Further data from other drug candidates (**Table 1**) in early phase trials is awaited to determine any divergent effects on gastrointestinal side effects.

A key initial concern of using glucagon within a co-agonist was unwanted hyperglycaemia. So far, the co-agonist drugs which have reached phase 2 trials have demonstrated an improved glycaemic profile with chronic administration. There is recent evidence that hepatic glucagon receptor stimulation may improve insulin stimulated glucose disposal. In a series of acute studies using euglycemic clamps, Kim and colleagues demonstrated that a glucagon agonist IUB288 leads to improved glucose tolerance by augmenting insulin action with evidence of increased hepatic AKT phosphorylation (88). Low dose glucagon agonism may therefore enhance insulin sensitivity which is in keeping with pre-prandial physiology in the fasted setting where the body is prepared to metabolise essential nutrients.

With chronic administration of glucagon-containing co-agonists, the catabolism of lean mass (i.e. protein and amino acids) becomes an important consideration. Direct evidence for the effect of glucagon on lean mass is seen from clinical situations of glucagon excess, in the glucagonoma syndrome (89). Despite the demonstrable importance of surveillance of lean mass during testing of obesity drug candidates, it is common for only fat mass loss or total body weight loss to be measured or presented in pre-clinical and early clinical trials. Furthermore, plasma amino acid levels are not routinely measured or reported. Exceptionally, in a Phase 2a study of cotadutide, individual plasma amino acid profiles were reported; after 49 days of treatment a significant reduction in plasma alanine was observed (84). However, with stronger glucagon receptor stimulation, it would be important for further pre-clinical studies to characterise the effects of lead drug candidates, on plasma amino acids and lean mass. Furthermore, with the increasing prominence of sarcopenic obesity, preserving lean mass in any weight loss strategy is important and this will be an area of increasing clinical and research interest.

## Future Perspectives/Conclusions

In the search for an anti-obesity pharmacotherapeutic which can rival the weight loss effects of bariatric surgery, research and development of gut hormone co-agonists is gaining momentum. GLP-1/glucagon co-agonists such as cotadutide and efinopegdutide offer the promise of increased efficacy whilst minimising side effects. Furthermore, with enhanced glucagon action the GLP-1/glucagon co-agonist has the advantage of being tailored to treat NAFLD directly.

**Figure 2:**
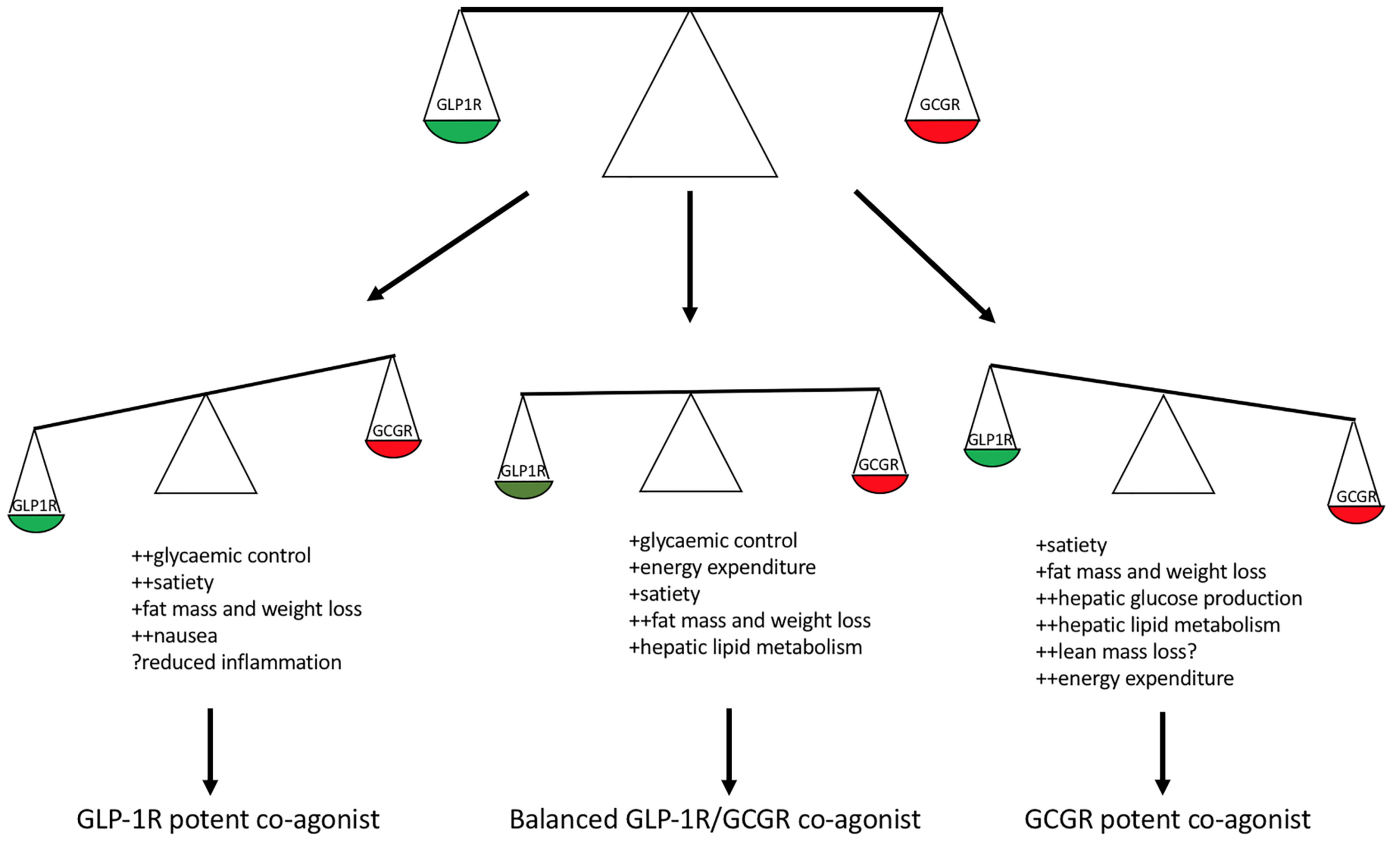
Balance of GLP-1R and GCGR potency within a co-agonist and predicted clinical effects. Glucagon receptor potent co-agonists predicted to result in enhanced weight loss due to increased energy expenditure however possible loss of lean mass and increased hepatic glucose production. GLP-1R potent co-agonists predicted to confer enhanced weight loss and glycaemic control with risk of gastrointestinal side effects. A balanced GLP-1R/GCGR co-agonist with respect to *in vitro* cAMP stimulation predicted to enhance glycaemic control and healthy fat mass loss.

However, the field is not staying still, and recently published data from the high-dose semaglutide STEP Phase 3 trials (44) and the SURPASS Phase 3 trials of the GLP-1/glucose-dependent insulinotropic peptide (GIP) co-agonist tirzepatide (90) have set the efficacy bar high in terms of weight loss and glycaemic improvement. We therefore conclude that before the place of GLP-1/glucagon co-agonists within the therapeutic armamentarium can be defined, future research efforts need to address the following outstanding questions:

1. What is the optimal balance and receptor potency of GLP-1 and glucagon in a long-acting co-agonist, to minimise adverse effects and to optimise efficacy (**Figure 2**)?
2. Does GLP-1/glucagon co-agonism offer long-lasting and enhanced efficacy for lowering blood glucose over that of GLP-1 analogues alone?
3. What are the optimal drug characteristics of a GLP-1/glucagon co-agonist for the treatment of NAFLD *via* hepatic GCGR agonism?
4. Are there long-term effects on lean mass with the co-agonists, and if so, can this be prevented?
5. Will the co-agonists inherit the favourable effects of GLP-1 analogues on prevention of cardiovascular events and progression of kidney disease?

## Author Contributions

All authors contributed to the article and approved the submitted version.

## Funding

DCDH is funded by a Medical Research Council Clinical Research Training Fellowship (MR/S02171X/1). MLV is supported by the NIHR Biomedical Research Centre.

## Conflict of Interest

TMMT is a shareholder and consultant for Zihipp Ltd., which is developing gut hormone analogues for treatment of metabolic disease.

The remaining authors declare that the research was conducted in the absence of any commercial or financial relationships that could be construed as a potential conflict of interest.

## Publisher’s Note

All claims expressed in this article are solely those of the authors and do not necessarily represent those of their affiliated organizations, or those of the publisher, the editors and the reviewers. Any product that may be evaluated in this article, or claim that may be made by its manufacturer, is not guaranteed or endorsed by the publisher.
